# Detecting heart stress using NT-proBNP in patients with type 2 diabetes mellitus and hypertension or high-normal blood pressure: a cross-sectional multicentric study

**DOI:** 10.1186/s12933-024-02391-z

**Published:** 2024-08-12

**Authors:** Matteo Landolfo, Francesco Spannella, Federico Giulietti, Beatrice Ortensi, Lucia Stella, Maria A. Carlucci, Roberta Galeazzi, Federica Turchi, Maria P. Luconi, Roberto Zampa, Sofia Cecchi, Elena Tortato, Massimiliano Petrelli, Riccardo Sarzani

**Affiliations:** 1 Internal Medicine and Geriatrics, IRCCS INRCA, Ancona, Italy; 2 Department of Clinical and Molecular Sciences, Politecnica delle Marche University, Ancona, Italy; 3 Unit of Diabetology– Endocrinology and Metabolic Diseases, AST Pesaro-Urbino, Urbino, Italy; 4 Clinic of Laboratory and Precision Medicine, IRCCS INRCA, Ancona, Italy; 5 Metabolic Diseases and Diabetology, IRCCS INRCA, Ancona, Italy; 6 Clinic of Endocrinology and Metabolic Diseases, Azienda Ospedaliero Universitaria delle Marche, Ancona, Italy

**Keywords:** NT-proBNP, Type 2 diabetes mellitus, Hypertension, Heart stress, GLP1-RA, SGLT2i

## Abstract

**Background:**

We evaluated the prevalence of “heart stress” (HS) based on NT-proBNP cut-points proposed by the 2023 Consensus of the Heart Failure Association (HFA) of the European Society of Cardiology (ESC) in asymptomatic patients with T2DM and hypertension or high-normal blood pressure (BP) eligible for SGLT2 inhibitors (SGLT2i) and/or GLP-1 receptor agonists (GLP1-RA), drugs with proven benefits on reducing the incidence of HF, hospitalizations, cardiovascular events and mortality.

**Methods:**

A cross-sectional multicentric study was conducted on 192 consecutive outpatients, aged ≥ 55 years, with hypertension or high-normal BP, referred to three diabetology units. NT-proBNP was collected before starting new anti-diabetic therapy. Patients with known HF were excluded, and participants were classified based on the age-adjusted NT-proBNP cut-points.

**Results:**

Mean age: 70.3 ± 7.8 years (67.5% males). Patients with obesity (BMI ≥ 30 Kg/m^2^): 63.8%. Median NT-proBNP: 96.0 (38.8–213.0) pg/mL. Prevalence of chronic kidney disease (CKD, eGFR < 60 mL/min/1.73m^2^): 32.1%. Mean arterial BP: 138.5/77.0 ± 15.8/9.9 mmHg. The NT-proBNP values, according to the proposed age-adjusted cut-points, classified 28.6% of patients as “HS likely” (organize elective echocardiography and specialist evaluation), 43.2% as “HS not likely” (a grey area, repeat NT-proBNP at six months) and 28.2% as “very unlikely HS” (repeat NT-proBNP at one year). The presence of CKD and the number of anti-hypertensive drugs, but not glycemic parameters, were independently associated with HS.

**Conclusions:**

According to NT-proBNP, over a quarter of T2DM patients with hypertension/high-normal BP, among those eligible for SGLT2i and/or GLP1-RA, were already at risk of cardiac damage, even subclinical. Most would receive an indication to echocardiogram and be referred to a specialist, allowing the early implementation of effective strategies to prevent or delay the progression to advanced stages of cardiac disease and overt HF.

## Introduction

Patients with type 2 diabetes mellitus (T2DM) and arterial hypertension are often overweight or obese (OW/OB) and have a higher risk of developing heart diseases up to any ejection fraction type of heart failure (HF) [1, 2]. Detection of patients with cardiovascular (CV) comorbidities at any risk for HF or those with subclinical HF would allow earlier implementation of effective strategies to prevent or delay the progression to overt and advanced stages of HF, such as optimizing the use of renin-angiotensin-aldosterone system (RAAS) inhibitors for blood pressure (BP) control or earlier initiation of other therapies with more recently proven ability to prevent HF progression. Indeed, many of these patients should receive GLP-1 receptor agonists (GLP1-RA) and SGLT2 inhibitors (SGLT2i) [3] after landmarking trials with both drug classes have proved considerable benefits in terms of reduction of HF incidence and hospitalization, major cardiovascular (CV) and renal events and CV mortality [4–7].

However, the implementation of available strategies to detect asymptomatic HF has been suboptimal in clinical practice, reducing the opportunities for more widespread awareness among patients and a more pragmatic application of beneficial therapies in such individuals. Although echocardiography might identify signs of maladaptive left ventricular remodeling, its routine use as a large-scale screening examination is not feasible and has not been considered cost-effective. It thus has not been systematically recommended for asymptomatic individuals, including those with T2DM. On the other hand, adding relatively inexpensive biomarker testing, such as natriuretic peptides (NPs), as part of the standard of care may help refine CV and HF risk prediction in T2DM individuals [8–10].

Based on extensive and solid clinical evidence, a recent Consensus Statement of the Heart Failure Association (HFA) of the European Society of Cardiology (ESC) [11] detailed the importance of assessing the levels of cardiac biomarker N-terminal-pro B-type Natriuretic Peptide (NT-proBNP) to evaluate T2DM patients at risk of HF in pre-failure or “heart stress” (HS), a new term introduced to identify asymptomatic individuals with risk factors and elevated plasma NPs, irrespective of the presence or absence of structural heart disease or cardiac dysfunction. Patients with T2DM serve as an example of this concept, as they often appear to have a structurally and functionally normal heart on imaging but with increased concentrations of NT-proBNP that predict an increased risk of developing HF [12]. However, despite NT-proBNP measuring should be part of an updated “Good Clinical Practice” (GCP), this biomarker is rarely (or even never) tested in real-life daily clinical practice, even in T2DM patients often affected by multiple comorbidities, such as older age, obesity and arterial hypertension.

The main objective of this study was to assess NT-proBNP levels to evaluate the prevalence of HS and its associated factors in outpatients affected by T2DM and arterial hypertension or high-normal BP who were eligible for SGLT2i and/or GLP1-RA treatment. For completeness, we also evaluated the prevalence of overt HF risk, always according to the NT-proBNP cut-points proposed by the HFA of the ESC, as a secondary outcome.

## Methods

### Study population and design

We performed an observational multicentric cross-sectional study on 192 consecutive T2DM outpatients referred to the Diabetology and Metabolic Diseases Centers of the IRCCS INRCA (Ancona, Italy), Clinic of Endocrinology and Metabolic Diseases of Azienda Ospedaliero Universitaria delle Marche (Ancona, Italy) and AST Pesaro-Urbino (Urbino, Italy) from May 2023 to April 2024. Inclusion criteria were the following: age ≥ 55 years, a history of arterial hypertension or high-normal BP (defined as the presence of anti-hypertensive treatment or office BP ≥ 130/85 mmHg on repeated and accurate measurements during medical visits [13]), an established diagnosis of T2DM, and clinical eligibility for treatment with GLP1-RA and/or SGLT2i [14] alone or on top of previous antidiabetics and lifestyle interventions. We included patients with high-normal BP in our study because these BP levels may also lead to cardiac remodeling over time [15, 16]. Exclusion criteria were the following: previous documented diagnosis of cardiac organ damage or HF, history of coronary artery disease or known cardiopathy, end-stage renal disease (ESRD), defined by an estimated glomerular filtration rate (eGFR using CKD-EPI creatinine equation) < 15 mL/min/1.73m^2^ or dialysis, advanced cancer or other terminal conditions (decompensated cirrhosis, severe dementia, bed-rest syndrome), any non-cardiac clinical conditions that could influence plasma value of laboratory parameters of interest, such as current liver cirrhosis or acute kidney injury (AKI). In this population, we evaluated the prevalence of HS and subclinical HF risk by measuring plasma NT-proBNP according to the recent 2023 Consensus Statement of the HFA of the ESC [11]. Before starting such additional pharmacological treatments, demographic, anthropometric, clinical and laboratory data were collected as part of GCP, including plasma NT-proBNP levels. Concerning CV and metabolic comorbidities, dyslipidemia was defined by the presence of lipid-lowering therapy (LLT) or low-density lipoprotein cholesterol (LDL-C) levels, calculated using the Friedewald equation modified by Martin et al. [17], above 70 or 55 mg/dL, according to the individual CV risk based on 2021 ESC Guidelines on Cardiovascular Disease Prevention in Clinical Practice [18]. Overweight and obesity were defined by a body mass index (BMI) ≥ 25 and ≥ 30 Kg/m^2^, respectively. Smoking habit was defined as the current or previous smoking of at least 100 cigarettes in a lifetime. Resistant hypertension was defined as uncontrolled hypertension despite the use of three anti-hypertensive medications of different classes, including a diuretic.

### NT-proBNP assay

NT-proBNP was measured using Elecsys proBNPII electrochemiluminescence immunoassay in a Cobas e601 immunoassay Roche analyzer. This assay contains two monoclonal antibodies that recognize epitopes in the N-terminal part (1–76) of proBNP (1–108) [19].

### NT-proBNP cut-points for the screening of heart stress and heart failure

The enrolled patients were classified according to NT-proBNP cut-points proposed by the 2023 Consensus Statement of the HFA of the ESC to diagnose and manage HS and HF [11], as summarized in Table 1. Regarding HS, evaluated in asymptomatic and clinically stable T2DM outpatients without significant and potentially confounding comorbidities, the study population has been classified into three subgroups according to age-adjusted NT-proBNP cut-points: “HS very unlikely”, “HS not likely”, and “HS likely” [11]. Similarly, the same population was also classified according to the “HF likely” risk proposed by the same Consensus Statement for the secondary outcome (Table 1).

**Table 1:** NT-proBNP cut-points for heart stress and heart failure risk in outpatients, according to the 2023 Clinical Consensus of the HFA of the ESC

	NT-proBNP cut-points
Heart stress	
Heart stress very unlikely	≤ 50 pg/mL
Heart stress not likely	Grey zone
Heart stress likely	
< 50 years	≥ 75 pg/mL
50–74 years	≥ 150 pg/mL
≥ 75 years	≥ 300 pg/mL
Heart failure	
Heart failure very unlikely	≤ 125 pg/mL
Heart failure not likely	Grey zone
Heart failure likely	
< 50 years	≥ 125 pg/mL
50–74 years	≥ 250 pg/mL
≥ 75 years	≥ 500 pg/mL
Heart failure very-high risk	≥ 2000 pg/Ml

Adapted by Bayes-Genis A. et al. “Practical algorithms for early diagnosis of heart failure and heart stress using NT-proBNP: A clinical consensus statement from the Heart Failure Association of the ESC” [11]

NT-proBNP: N-terminal-pro-Brain Natriuretic Peptide

### Statistical analysis

Data were analyzed using the Statistical Package for Social Science version 21 (SPSS Inc., Chicago, Illinois, USA). The study’s primary outcome was to evaluate the prevalence of “HS likely” and its associated factors in the enrolled population. Continuous variables were checked for normality and expressed as mean ± standard deviation (SD) or median and interquartile range (IQR) if markedly skewed. Frequencies were expressed as percentages. Analysis of variance (ANOVA) and Kruskal–Wallis tests were used to evaluate differences between continuous variables. Correlations between continuous variables were assessed using Pearson’s and Spearmans’ tests. The triglycerides variable was naturally logarithmically transformed to normalize its distribution. Those variables with a significant association identified upon univariate analyses were tested within a stepwise logistic regression model (entry value = 0.05 and removal value = 0.10) to evaluate the factors independently associated with the outcome of interest. We used the “forward model” that starts to include variables by their order of significance. This method is usual in exploratory studies, where there is no certainty of what variables may influence the outcome.

## Results

**Fig. 1:**
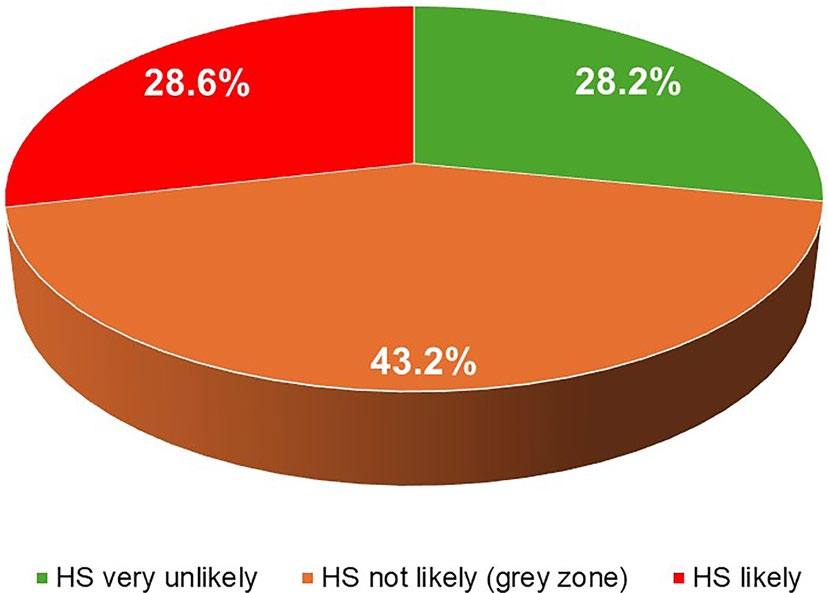
Prevalence of the different Heart Stress states in the study population. HS: heart stress

A total of 192 T2DM patients with hypertension or high-normal BP eligible for GLP1-RA (n° 83) or SGLT2i (n° 109) treatment were enrolled in the study. General characteristics of the entire study population and according to the HS status are described in Table 2. In the overall population with a mean age of 70.3 ± 7.9 years and male prevalence of 67.7%, most patients were affected by obesity, dyslipidemia and poor BP control, with a 32.1% prevalence of chronic kidney disease (CKD) [eGFR < 60 mL/min/1.73m^2^, by CKD-EPI equation]. Regarding NT-proBNP values, the overall median was 96.0 (38.8–213.0) pg/mL. Figure 1 shows the classification of the patients according to age-adjusted NT-proBNP cut-points. The higher the probability of HS, the greater the age of patients and the presence of renal dysfunction. Patients with “HS likely” tended to have lower diastolic BP, a higher prevalence of resistant hypertension, although not statistically significant, and lower levels of triglycerides. Regarding CV treatments, the higher the probability of HS, the greater the number of anti-hypertensive drugs taken, while the lower the metformin use at baseline (Table 2). No associations were found with T2DM duration or vascular, retinal and cerebral organ damage.

By Spearman’s test, NT-proBNP significantly and directly correlated with age (*r* = 0.526, *p* < 0.001), T2DM duration (*r* = 0.179, *p* = 0.015) and pulse pressure (*r* = 0.166, *p* = 0.031), while inversely correlated with eGFR (*r* = − 0.368, *p* < 0.001), BMI (*r* = − 0.202, *p* = 0.023), waist circumference (*r* = − 0.160, *p* = 0.028) and TyG index (*r* = − 0.234, *p* = 0.013).

Regarding the HF likely risk, NT-proBNP levels classified 60.9% (*n* = 117) as “HF very unlikely”, 22.4% (*n* = 43) as “HF not likely”, 15.1% (*n* = 29) as “HF likely”, and 1.6% (*n* = 3) as “HF very-high risk”.

**Table 2:** General characteristics of the overall study population and according to the heart stress status

	Overall population (*n* = 192)	HS very unlikely (*n* = 54)	HS not likely (grey zone) (*n* = 83)	HS likely (*n* = 55)	*p * values*
Demographics and anthropometrics					
Age (years)	70.3 ± 7.9	65.9 ± 7.2	70.8 ± 7.2	73.8 ± 7.6	< 0.001
Sex (male)	67.7%	74.1%	60.2%	72.7%	0.153
BMI (Kg/m^2^)	32.9 ± 6.0	35.0 ± 6.3	31.8 ± 5.9	32.3 ± 5.1	0.006
Waist circumference (cm)	112.4 ± 13.6	116.9 ± 13.4	109.7 ± 13.6	111.9 ± 12.9	0.010
CKD (eGFR < 60 ml/min/1.73m^2^)	32.1%	21.6%	24.1%	51.1%	0.004
Obese (BMI > 30 Kg/m^2^)	63.8%	73.6%	58.5%	62.3%	0.198
Systolic BP (mmHg)	138.5 ± 15.8	138.2 ± 16.0	139.0 ± 15.3	138.0 ± 16.6	0.938
Diastolic BP (mmHg)	77.0 ± 9.9	79.9 ± 11.2	76.1 ± 9.1	75.6 ± 9.2	0.058
Pulse pressure (mmHg)	61.5 ± 14.1	58.3 ± 11.8	62.9 ± 14.8	62.5 ± 14.8	0.181
Uncontrolled hypertension	78.1%	83.0%	79.5%	71.4%	0.366
Resistant hypertension	13.0%	3.7%	16.9%	16.4%	0.056
Smoking habit	33.3%	35.2%	28.9%	38.2%	0.498
Dyslipidemia	87.0%	81.5%	89.2%	89.1%	0.367
T2DM duration (years)	12.7 ± 9.9	10.4 ± 8.7	14.2 ± 10.3	12.8 ± 10.1	0.083
Diabetic retinopathy	17.9%	11.6%	17.7%	23.9%	0.318
Peripheral artery disease	61.9%	57.5%	60.6%	68.3%	0.581
Cerebrovascular disease	6.1%	8.2%	5.0%	5.8%	0.623
Laboratory parameters					
eGFR (mL/min/1.73m^2^)	73.9 ± 24.0	84.0 ± 25.1	74.9 ± 22.3	64.2 ± 22.0	0.001
NT-proBNP (pg/mL)	96.0 (38.8–213.0)	27.0 (18.5–35.3)	95.0 (64.0-124.4)	456.0 (216.1–1262.0)	< 0.001
Total cholesterol (mg/dL)	157.9 ± 36.9	165.4 ± 36.5	157.7 ± 39.2	152.6 ± 34.2	0.349
HDL-C (mg/dL)	46.7 ± 10.5	48.1 ± 9.0	45.3 ± 9.8	47.3 ± 12.5	0.458
LDL-C (mg/dL)	81.5 ± 32.4	88.0 ± 30.4	79.6 ± 35.5	78.5 ± 30.1	0.425
Triglycerides (mg/dL)	130.5 (98.8–190.3)	147.0 (109.0–195.0)	145.0 (101.0–207.0 )	114.5 (92.0–154.5)	0.024
Glycaemia (mg/dL)	146.5 (126.0–171.5)	147.0 (135.0–181.0)	146.0 (125.0–171.0)	147.0 (123.5–168.5)	0.646
HbA1c (mmol/mol)	59.0 (53.0–67.0)	61.0 (54.1–72.2)	60.0 (53.0–68.0)	58.0 (50.0–63.9)	0.187
Albuminuria (≥ 30 mg/g creatinin)	40.6%	48.3%	40.4%	32.0%	0.478
TyG index	9.2 (8.9–9.5)	9.2 (9.0-9.5)	9.3 (8.9–9.7)	9.1 (8.8–9.3)	0.104
Cardiovascular therapy					
LLT	78.1%	74.1%	77.1%	83.6%	0.462
Metformin	76.0%	90.7%	77.1%	60.0%	0.001
Insulin therapy	14.1%	11.1%	15.7%	14.5%	0.750
ACEi	26.6%	20.4%	27.7%	30.9%	0.438
ARB	48.4%	46.3%	45.8%	54.5%	0.561
Diuretic	44.8%	33.3%	47.0%	52.7%	0.109
Calcium antagonist	42.2%	31.5%	42.2%	52.7%	0.080
MRA	5.7%	0.0%	6.0%	10.9%	0.015
Beta-blocker	38.0%	20.4%	39.8%	52.7%	0.002
Alpha-blocker	7.3%	3.7%	3.6%	7.3%	0.011
Number of anti-hypertensives	2.1 ± 1.3	1.6 ± 1.2	2.1 ± 1.2	2.7 ± 1.2	< 0.001

HS: Heart Stress; BMI: Body Mass Index; CKD: Chronic Kidney Disease; BP: blood pressure; T2DM: type 2 diabetes mellitus; eGFR: estimated glomerular filtration rate; NT-proBNP: N-terminal pro-B-type Natriuretic Peptide; HDL-C: high-density lipoprotein cholesterol; LDL-C: low-density lipoprotein cholesterol; HbA1c: glycated haemoglobin; TyG: triglyceride-glucose index; LLT: lipid-lowering therapy; ACEi: angiotensin-converting enzyme inhibitor; ARB: angiotensin-receptor blocker; MRA: mineralocorticoid receptor antagonist

*p-values refer to comparing the three “heart stress” statuses. Pulse pressure was defined as the difference between systolic BP and diastolic BP, and was considered as a crude surrogate of arterial stiffness. Uncontrolled hypertension was defined as BP ≥ 130/80 mmHg; Resistant hypertension was defined as uncontrolled hypertension despite the use of three anti-hypertensive medications of different classes, including a diuretic. Peripheral artery disease includes the presence of atherosclerotic plaques in the carotid artery and lower and upper extremity arteries. TyG index, a biomarker of insulin resistance, was calculated using the following formula: Ln [fasting triglycerides (mg/dL) × fasting plasma glucose (mg/dL)/2]

### Factors associated with heart stress

A stepwise logistic regression was performed to evaluate factors independently associated with HS. For this analysis, we grouped patients with “HS very unlikely” and “HS not likely”, the two groups with recommendations that are more similar to each other (see below) and have less impact on patient management. The statistically significant variables in the univariate analyses were used as independent variables [age, CKD, Ln(Triglycerides), eGFR, metformin, MRA, beta-blocker, alpha-blocker, number of anti-hypertensive drugs]. Among these factors, only CKD [Wald = 6.3; OR = 2.8 (95% CI 1.3–6.2); *p* = 0.012] and the number of anti-hypertensive medications [Wald = 10.7; OR = 1.8 (95% CI 1.3–2.5); *p* = 0.001] were included in the final model, showing a significant independent association with “HS likely” (Table 3).

**Table 3:** Probability of heart stress in the study population

	Univariate analysis		Multivariate analysis*	
	OR (95% CI)	*p*	OR (95% CI)	*p*
Age (years)	1.1 (1.0–1.1)	< 0.001	–	–
CKD (eGFR < 60 ml/min/1.73m^2^)	3.5 (1.6–7.4)	0.001	2.8 (1.3–6.2)	0.012
eGFR (ml/min/1.73m^2^)	0.9 (0.9–1.0)	0.002	–	–
Ln(Tryglicerides)	0.3 (0.1–0.7)	0.006	–	–
Metformin	0.3 (0.2–0.6)	0.001	–	–
MRA	3.2 (0.9–11.1)	0.051	–	–
Beta-blocker	2.4 (1.2–4.5)	0.009	–	–
Alpha-blocker	5.2 (1.6–16.2)	0.005	–	–
Number of anti-hypertensives	1.8 (1.3–2.3)	< 0.001	1.8 (1.3–2.5)	0.001

CKD: Chronic Kidney Disease; eGFR: estimated glomerular filtration rate; MRA: mineralocorticoid receptor antagonist

*Stepwise logistic regression with the variables with significant association at univariate analyses, as independent variables

## Discussion

In our study, more than a quarter of patients with T2DM and high BP showed an underlying HS likely, which would not have been detected without the NT-proBNP dosage, a very relevant finding in this type of patient. Risk factors that emerged independently associated with this condition were the presence of chronic kidney disease and the number of anti-hypertensive drugs taken.

Core evidence from epidemiological studies reported an increasing incidence rate of HF among people with diabetes, even after adjustment for age and other comorbidities. HF is becoming the first presenting CV complication in T2DM individuals [20]. Our population of older T2DM patients showed a relatively high prevalence of CV, renal and metabolic comorbidities, establishing an even more fertile terrain for acute major cardiac events and worsening heart diseases, for which HF represents the inevitable consequence. In this clinical context, progressive cardiac dysfunction reflects complex interactions between ageing, adiposity and CV risk factors-related deleterious mechanisms that affect myocardial metabolism, structure and efficiency [21]. Consequently, clinical overt HF represents the epiphenomenon of a continuum of such cardiac abnormalities. The 2021 multi-society consensus statement on “Universal Definition and Classification of Heart Failure” places patients with hypertension, atherosclerotic CV disease, diabetes and obesity already in stage A HF (at risk for HF), even in the absence of current or prior symptoms or signs of HF and structural cardiac changes or elevated biomarkers of HF [22]. The correct management of CV-modifiable risk factors through lifestyle and pharmacological interventions is the cornerstone of managing stage A HF and can slow the progression to overt HF. Unfortunately, current trends suggest that control of modifiable risk factors is poor in patients with diabetes [23]. Our real-life sample reflects this concern, with the high prevalence of uncontrolled BP, high mean LDL-C and a quarter of T2DM hypertensive patients not taking a renin-angiotensin-aldosterone system inhibitor (RAASi), the first choice drug for the treatment of arterial hypertension, especially in the presence of T2DM, for its proven cardio-renal protection [24].

Recently, renewed emphasis has been placed on the potential role of myocardial stress biomarkers, particularly NT-proBNP, in detecting T2DM individuals with early cardiac dysfunction and at the highest risk of HF progression [11, 25–27]. The cardiac biomarker-guided screening approach is already included as a class IIa recommendation in the 2017 American College of Cardiology/American Heart Association (ACC/AHA) HF Focused Update [28]. Accordingly, the latest 2023 Clinical Consensus of the HFA of the ESC recommended that NT-proBNP levels should be assessed whenever a patient is at risk of developing CV disease and HF to identify “heart stress”, a new condition sitting in between stages A and B HF and identifying asymptomatic individuals with risk factors and elevated plasma NT-proBNP, irrespective of the presence or absence of structural heart disease or cardiac dysfunction [11]. In our investigation, despite the lack of overt HF-related clinical manifestations (i.e., dyspnoea, pulmonary rales, etc.), 28.6% of the enrolled patients had NT-proBNP values compatible with an HS condition, and about 40% fell in the grey zone. Along with the novelty represented by specific NT-proBNP cut-points, it is noteworthy that dosing NT-proBNP in stable asymptomatic outpatients with CV comorbidities represents not only a mere HF risk evaluation but strongly impacts management strategies. Indeed, according to the HFA Clinical Consensus recommendations, all patients in the “HS likely” spectrum of NT-proBNP values should be evaluated with elective echocardiography and assessed by the HF team if cardiac dysfunction is found. In contrast, all patients with values compatible with “HS not likely” and “HF very unlikely” should at least enter follow-up and repeat NT-proBNP dosing after six months or one year, respectively. Similarly, also the 2024 updated American Diabetes Association (ADA) Standards of Care in Diabetes (section of Cardiovascular Disease and Risk Management) recommended echocardiography to identify stage B HF in asymptomatic individuals with diabetes and abnormal natriuretic peptide levels (class A) [29].

No previous studies investigated the prevalence of unknown HS using NT-proBNP as an early and asymptomatic first stage of heart disease in T2DM patients. However, estimates of how HF is spread in the T2DM population are available. A study on a casual older T2DM population, using echocardiography as a screening tool, found a prevalence of 27.7% of unknown symptomatic HF with 25.1% of diastolic dysfunction [30]. A recent systematic review [31] summarized the studies which explored the prevalence of different degrees of heart damage in adults ≥ 18 years with T2DM, using mainly echocardiographic data. Based on 65 studies on 25,729 individuals, it found that there is a large pre-clinical group with left ventricular diastolic dysfunction / HFpEF [43% (95% CI 37%, 50%) and 17% (95% CI 7%, 35%), respectively] in which cardiac disease progression could be halted by early recognition and treatment. As expected, a high risk of bias and high levels of heterogeneity due to the different diagnostic criteria over the years and the different characteristics of study populations limited this analysis [31]. A recent study used the NT-proBNP point-of-care measurement as a screening tool for HF in a population with T2DM and hypertension. Although the mean age was slightly lower than ours, the authors found NT-proBNP levels ≥ 125 pg/ml in 39.4% of the study population [32].

In addition to the HS status, we also tested HF likely according to the age-adjusted NT-proBNP cut-points proposed by the 2023 Clinical Consensus of the HFA of the ESC for outpatients suspected of de novo HF. Considering the higher NT-proBNP cut-points for HF compared to HS, we found that 16.7% of patients had NT-proBNP values ​​compatible with at least HF likely. Considering the non-specificity of HF symptoms, including asthenia and dyspnea, that are not rare in obese T2DM patients with other CV risk factors, these data further reinforce the relevance of this biomarker in clinical practice.

In our study, unsurprisingly, older patients had a higher prevalence of HS, given that ageing acts by “giving time” to the other CV risk factors to produce vascular and cardiac damage [33]. In addition to age, we found that the number of anti-hypertensive drugs taken and the renal function are likely risk factors for HS. In contrast, no independent association was found with the glycemic parameters evaluated, such as baseline glycaemia and HbA1C. This last finding could be due to several hypotheses. It could reflect a pivotal role of the BP burden compared to alterations in glucose metabolism on cardiac organ damage, in which pressure and volume overload, especially in obese patients, leads to a modification of the left ventricular structure and function [34, 35]. On the other hand, the absence of association with glycemic parameters, as well as with BP values and control, could be due to the study’s cross-sectional design, which provides only a screenshot of the patient’s condition (we found only a direct correlation at unadjusted univariate analysis between NT-proBNP levels and T2DM duration in our sample). Instead, the number of anti-hypertensive drugs could better reflect the patient’s BP severity and burden over time.

A wide study of epidemiological cohorts showed that a biomarker-based risk score including NT-proBNP (cut-point ≥ 125 pg/ml) together with high-sensitivity cardiac troponin-T, high-sensitivity C-reactive protein and electrocardiographic-based LVH based on Sokolow-Lyon criteria, was able to predict diabetic and pre-diabetic patients at higher 5- and 10-year HF risk [36]. The same study also found that patients with higher scores and a higher risk of HF onset would benefit the most from SGLT2i therapy regarding incident HF prevention [36]. Our analysis focused on patients eligible for SGLT2i and GLP1-RA. Due to their multiple beneficial effects (renal protection, improvement of myocardial energy metabolism, positive impact on anaemia, etc.) and their hybrid diuretic action [37], several studies have confirmed the efficacy of SGLT2i, especially in preventing HF incidence and hospitalization. The risk reduction of this outcome resulted the more consistent across the trials examined by a recent metanalysis (HR, 0.68; 95% CI 0.61–0.76) [38]. Thus, from the clinical perspective, another relevant issue concerning the NT-proBNP dosing in the diabetic population is related to its usefulness in identifying those patients who would benefit the most from this drug class before initiating treatment, also in the wake of data on biomarker-guided therapy that controversially and limited to the chronic HF setting was demonstrated to reduce CV outcomes, especially when pharmacological treatments with first-line drugs reached target doses [39, 40]. In the RCTs, chronic administration of SGLT2i was associated with a significant and progressive reduction of NT-proBNP at each timepoint examined, independently of the molecule tested [41]. However, it was observed that a lower incidence of major adverse cardiac events (MACEs) was achieved regardless of their baseline NT-proBNP value [42].

Unlike SGLT2i, which can now be administered despite diabetic status in HF and CKD patients with albuminuria, GLP1-RA, which has a more significant influence on glycaemic control, can currently be prescribed in T2DM and/or obese patients only. Indeed, it is not yet indicated for HF, despite growing evidence of improvement in symptoms and quality of life is available regarding HF with preserved ejection fraction (HFpEF), the one most closely linked to adiposity and dysmetabolism [43, 44]. GLP1-RAs have also been proven to reduce NT-proBNP across all trials significantly [41]. Unlike SGLT2i, whenever compared with placebo, GLP1-RAs do not seem to have the same behaviour across all the EF spectrum. Indeed, they do not seem to significantly reduce the composite of HF hospitalization or CV death and all-cause mortality in patients with HF history, as in patients without HF history (HR 0.84, 95% CI: 0.76–0.92 and HR 0.85, 95% CI: 0.79–0.92, respectively) [45]. A recent meta-analysis of two RCTs questioned the potential risk of HF hospitalization following the introduction of GLP1-RA (liraglutide and exenatide) in T2DM patients with overt HF in the setting of HFrEF, finding a 50% augmented risk (OR 1.49, 95% CI 1.05–2.10, *p* = 0.02; I2 = 0%) [46]. These results prompted the same authors to suggest performing active HF screening by employing NP dosage and careful evaluation of clinical history, physical examination and ultrasound imaging before initiating GLP1-RA. However, these meta-analyses include trials in which hospitalization and mortality were not the primary objective and may not be conclusive. While waiting for the findings of future trials focused on these major outcomes in both HFrEF and HFpEF, secure evidence shows how GLP1-RAs improve symptoms and quality of life in obese subjects with HFpEF. Therefore, T2DM people without HF should expect a reduced risk of HF incidence due to the protection of CV events [47, 48].

### Study limits

This study has several limitations that need to be addressed. The small sample size and the cross-sectional design are the primary limits, not allowing us to extend our findings to younger populations or patients not eligible for therapy with SGLT2i and/or GLP1-RA. However, the study population was enrolled in three different centres, reflecting the population encountered in “real-life” daily clinical practice. It can be configured as an exploratory study to launch future studies/registries on an epidemiological scale. Moreover, this study is the first to have evaluated the prevalence and associated factors for HS, a concept only recently introduced by the ESC, in T2DM patients with concomitant hypertension/high-normal BP and T2DM, eligible for SGLT2i and GLP1-RA, drugs that have been found to exert several benefits on cardiac dysfunction. In our paper, the echocardiographic data of the patients were not systematically available and therefore described. Our primary intention was to raise awareness of the potential usefulness of the readily available and low-cost NT-proBNP determination in the specific setting of T2DM hypertensive patients. Knowing the NT-proBNP values of my patient can already change his clinical management, as previously reported; therefore, knowing or not the presence of cardiac structural alterations is not one of the objectives of this study.

## Conclusions

High BP and T2DM lead to an increased risk of cardiac remodeling and overt HF over time. Large-scale screening with cardiac biomarkers in these patients could allow early and appropriate clinical phenotyping and lead to a more focused implementation of preventive measures based on lifestyle and pharmacological intervention. Future studies on broader populations should evaluate such an approach’s feasibility and clinical impact. Our analysis reveals a non-negligible prevalence of HS in this population, that would have gone unnoticed if not for the NT-proBNP dosage. BP burden and concomitant renal damage were found to be the most relevant risk factors for HS in our study, stressing the pivotal role of higher blood pressure and hypertension in determining cardiac damage in T2DM patients. Therefore, NT-proBNP helps identify hypertensive patients with T2DM at risk of cardiac damage, even subclinical, with an indication to perform an echocardiogram and refer to a specialist team, allowing the early implementation of effective strategies to prevent or delay the progression to overt and advanced stages of the cardiac disease.

## Data Availability

The data supporting this study’s findings are available from the corresponding author (FS) upon reasonable request.
